# Benchmarking gene set of gymnosperms for assessing genome and annotation completeness in BUSCO

**DOI:** 10.1093/hr/uhad165

**Published:** 2023-08-17

**Authors:** Jun-Jie Wu, Yu-Wei Han, Chen-Feng Lin, Jing Cai, Yun-Peng Zhao

**Affiliations:** Systematic & Evolutionary Botany and Biodiversity Group, MOE Key Laboratory of Biosystem Homeostasis and Protection, College of Life Sciences, Zhejiang University, Hangzhou 310058, China; School of Ecology and Environment, Northwestern Polytechnical University, Xi’an 710129, China; Systematic & Evolutionary Botany and Biodiversity Group, MOE Key Laboratory of Biosystem Homeostasis and Protection, College of Life Sciences, Zhejiang University, Hangzhou 310058, China; School of Ecology and Environment, Northwestern Polytechnical University, Xi’an 710129, China; Systematic & Evolutionary Botany and Biodiversity Group, MOE Key Laboratory of Biosystem Homeostasis and Protection, College of Life Sciences, Zhejiang University, Hangzhou 310058, China

**Figure 1:**
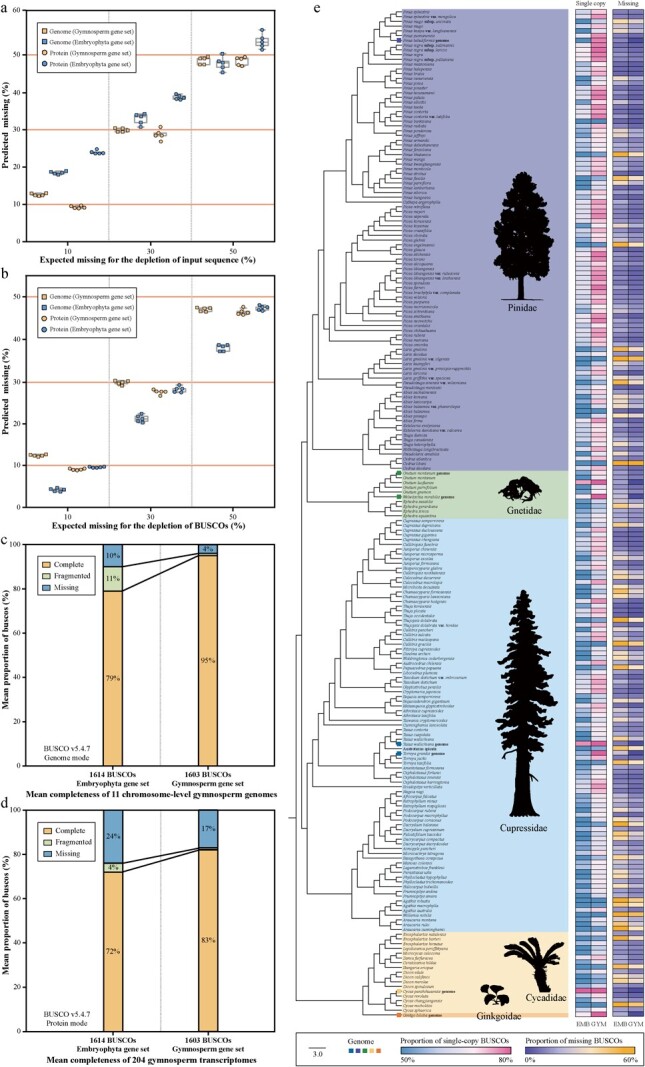
Comparison of the gymnosperm gene set with the embryophyte gene set. **a**, **b** Expected and predicted missing values for the input sequence (**a**) and BUSCOs (**b**) with different levels of depletion using the gymnosperm (yellow) and Embryophyta (blue) gene sets under genome (rectangle) and protein (circle) modes in BUSCO (version 5.4.7). Solid orange lines indicate the expected missing values. **c**, **d** Estimates of mean completeness of the 11 chromosome-level gymnosperm genomes (**c**) and 204 gymnosperm transcriptomes (**d**) using the gymnosperm and Embryophyta gene sets. **e** Species tree inferred from the 1603 gene trees based on the gymnosperm data set using ASTRAL. The rectangles on the species tree represent the gymnosperm genomes used in the gymnosperm gene set. Two heat maps show the proportion of complete and missing BUSCOS using the gymnosperm (GYM) and the Embryophyta (EMB) gene set in BUSCO (version 5.4.7); the color intensity indicates the proportion. Plant silhouettes of Ginkgoidae, Cycadidae, Cupressidae, Gnetidae, and Pinidae were obtained from PhyloPic (http://phylopic.org).

Dear Editor,

Gymnosperms, naked-seed plants, are phylogenetically sister to the flowering plants (angiosperms), of which the origin can be traced back to the Devonian period. Despite their flourishing diversity in the Mesozoic era, only ~1000 living species of gymnosperms exist in the modern flora (~0.4% of the number of angiosperms) [1]. However, they account for at least 39% of the world’s forests [2] and hold great economic and cultural importance in horticulture, medicine, and the timber industry [3]. Extant gymnosperms comprise five major lineages, i.e. Cycadidae, Ginkgoidae, Cupressidae, Pinidae, and Gnetidae, which provide an essential phylogenetic backbone for understanding plant evolution [4]. Unfortunately, obtaining high-quality genomes and annotations remains a major challenge for gymnosperm research due to their genomic characteristics, e.g. huge genome size, highly repetitive sequence content, and ultra-long introns. With advances in sequencing technology and assembly algorithms, an increasing number of gymnosperm species were sequenced. Nevertheless, when assessing genome assembly and annotation completeness, the dataset for land plants (embryophyta_odb10) used in BUSCO (Benchmarking Universal Single-Copy Orthologs) [5] is not applicable to gymnosperms due to the far evolutionary distance between the major lineages, especially between angiosperms and gymnosperms. As a result, the average genome completeness was estimated as low as 79% for 11 gymnosperm species (Fig. 1c). To address this problem, we developed and validated a BUSCO dataset of 1603 predefined single-copy genes (hereafter BUSCOs) dedicated to gymnosperms based on seven chromosome-level genomes representing all the five clades of gymnosperms. This benchmarking gene set of gymnosperms is available at https://github.com/jjwujay/Gymnosperm_odb10.

The current public BUSCO datasets were derived from the predefined single-copy orthologs in OrthoDB, a database providing orthologs identified across genomes [5]. Due to the paucity of gymnosperm sequences in OrthoDB v11, single-copy orthologs were identified in our study based on available gymnosperm genomes. As of 30 June 2023, 22 gymnosperms have been sequenced. Considering both the quality of the genome and the representation of major lineages, we selected those gymnosperm genomes that were assembled and anchored at the chromosome level. In addition, as the number of available genomes grows, it becomes increasingly challenging to identify reliable single-copy orthologs present in more taxa [6]. Therefore, after screening, we conservatively selected seven high-quality genomes from all the five major clades of gymnosperms (Fig. 1e). Although the number of gymnosperm genomes appeared small, the sampling rate of our gymnosperm gene set (~7/1000) was indeed remarkably higher than that of flowering plant datasets, such as eudicotyledons_odb10 (~ 40/190000) and liliopsida_odb10 (~ 18/20000). We then identified the orthogroups among the annotated proteins of these genomes using OrthoFinder [7] (version 2.5.1). By filtering the gene counts of the OrthoFinder results (gene copy number = 1 or 2; total gene number per ortholog ≤9), we obtained a raw gene set consisting of 552 single-copy orthologs and 1051 low-copy orthologs. The latter were further partitioned into the former using OrthoSNAP [6] (version 0.0.1), resulting in a total of 1603 single-copy orthologs. To assure consistency with the original dataset format, we generated the required profiles of 1603 predefined single-copy genes using both msa2prfl.pl in Augustus [8] (version 3.4.0) and hmmbuild and hmmemit in HMMER [9] (version 3.1b2). The HMMER cut-off for each ortholog was set as 90% of the value of the lowest score in the results of hmmseach against itself, and the length range was set to the mean with twice the standard deviation. Given the ultra-long length of introns and genes in gymnosperms, we set the maximum values as 2200 and 1600 kb, respectively, according to the current longest gene (~2100 kb) and intron (~1500 kb) length in gymnosperms.

To assess the reliability of the gymnosperm benchmark gene set, we generated multiple data sets for BUSCO analyses by randomly deleting different proportions of input sequences. Briefly, we randomly removed 10/30/50% of the annotated protein sequences or the single-copy orthologs of the tested species, and masked the corresponding regions of the genome by coding them as missing information (N). The modes of genome and protein in BUSCO (version 5.4.7) [5] were used to estimate the proportion of missing BUSCOs, and five replicates were set for each level of depletion. We found a high level of concordance between the estimated proportions of missing data in various test sets and the expected values when utilizing our gymnosperm benchmark gene set (average offset in genome mode = 1.7%; average offset in protein mode = 1.9%), in contrast to the substantial variation of the predictions derived from the Embryophyta dataset (average offset in genome mode = 6.8%; average offset in protein mode = 5.3%; Fig. 1a and b). These results showcased that our gymnosperm benchmark gene set was more suitable for gymnosperms, highlighting the importance of lineage-specific data sets. We also assessed the completeness of all 11 currently available chromosome-level gymnosperm genomes (average completeness = 95%) and the 204 gymnosperm transcriptomes (~20% of living gymnosperms, average completeness = 82%) using our dataset (Fig. 1c and d). Our results revealed that, compared with the Embryophyta dataset, our dataset notably increased the proportions of complete BUSCOs of gymnosperm genomes (average increase in completeness = 16%) and transcriptomes (average increase in completeness = 11%) as well as remarkably reducing the missing proportions (Fig. 1e). Phylogenetic reconstruction based on the single-copy genes identified by BUSCO using ASTRAL (version 5.7.8) generated a gymnosperm tree (Fig. 1e) consistent with those in previous studies [10]. In conclusion, we developed and validated the first robust benchmarking gene set of gymnosperms for assessing genome and annotation completeness in BUSCO. This benchmarking gene set can serve as a vital resource for scientific researchers on gymnosperm genomes, helping to fill the gap of high-quality, complete genomes in the phylogeny of land plants.
